# Gynæcological and Obstetrical Society of Baltimore

**Published:** 1889-02

**Authors:** 


*Regular Meeting, December 11, 1888.*

THE PRESIDENT, DR. THOS. OPIE, IN THE CHAIR.

DR. G. W. MILTENBERGER read a paper on

### TETANOID CONTRACTION OF THE UTERUS.

On the evening of October 19th, 1887, I was called by my friend Dr. H. M. Wilson, a most skilled and experienced accoucheur, to see with him a case of labor, some six or eight squares from my office. From the statement of the messenger I expected to meet with a case of difficult delivery in a primipara, but on entering the chamber of Mrs.—— I was horrified, on meeting the doctor, to be told that his patient was dying, and to see a large, finely developed woman, 27 or 28 years of age, in the very agony of death. Total unconsciousness, two or three gasping respirations, a faint beat or two of the heart, when all was still and the scene closed. I immediately listened for the sounds of the fœtal heart, but not a throb responded; mother and child were both dead. The doctor and the husband requested me to at once deliver the child, and learning that the doctor had already made a fruitless endeavor with Simpson's and later Tarnier's forceps, and knowing thoroughly his experience and skill, and being told that with the instrument he could bring the vortex down to the vulva, but the moment traction was relieved the head flew back above the superior strait, I realized the inutility of their employment, and proceeded to peform version.

Upon introducing the right hand, supporting the fundus uteri with the left, I found a vertex presentation, R. O. I. A. position, head above the superior strait. The os was perfectly soft and dilatable, not offering the slightest resistance, head movable, cervix soft and relaxed, not thinned nor distended, no strain upon the structure whatever. With the external hand I found nearly up to the umbilicus of the mother, a deep furrow upon the external surface of the uterus corresponding to the so-called ring of Bandl, the contraction ring of Schroeder, the retraction ring of Lusk, the organ above this line being firmly contracted and retracted. The internal hand, readily passing the head, met with a ring or constriction, corresponding to the external furrow, entirely too high for the internal os uteri, surrounding most closely and tenaciously the neck of the child, the head below in the soft, relaxed and not distended or stretched lower segment, the trunk in the contracted and retracted portion above. This band or constriction was as firm and resistant as a *rope of steel.* I could with the most determined effort force the points of one or two of the fingers between this and the neck, but for a time no further. I certainly never had felt anything like it before, but recognized the condition called by that eminent authority, R. P. Harris, Philadelphia, "Tetanoid Falciform Contraction of the Uterus," and Dr. Hosmer and Dr. Thos. C. Smith, of Washington, D. C., "Ante-partum Hour-glass Contraction of the Uterus," in the latter's valuable résumé of these cases in the *American Journal of Obstetrics*, Vol. XV, November, 1882. I for a time feared I would be foiled, but after a most determined effort I finally succeeded in passing it and ultimately reaching the foot. Still the resistance did not entirely yield, and it was only after further powerful and persistent effort that the constriction yielded and I was able to turn and extract. I am convinced that I could not without injury have succeeded during life. The placenta was then removed without further difficulty.

I then learned from Dr. Wilson the previous history of the case. Five days previously the membranes had ruptured and waters had been discharged. On the morning of the 19th he was called at 6 A. M., found pains of first stage, soft parts of pelvis relaxed and well lubricated, vertex presenting R. O. I. A. position, as far as he could determine, the external os still closed. Upon returning in two hours, at 8 A.M., os was dilated to size of a dime, edges soft and thin, and he then remained with her uninterruptedly until the fatal close, so that she was constantly and closely watched, and every possible attention and care bestowed. Everything seemed to progress favorably, with occasional but not inordinate nausea and vomiting. The advance was slow and gradual, and about 5.30 P.M. the os was sufficiently dilatable to apply the forceps, which was cautiously and carefully done. Simpson's were first used, there being no difficulty in their adjustment. No advance, however, could be effected and they were removed and Tarnier's applied. With the latter he could cause the caput to appear at the vulva, but when the pressure was relaxed it would instantly recede to its former position.

It was at this juncture I was sent for, and the doctor finding her pulse failing resorted to all means of resuscitation, but she died in about 30 or 40 minutes. The patient had had chloroform administered at the height of her pains previously to the use of the forceps, but only to the obstetric degree, not to full anæsthesia or unconsciousness. With the application of the forceps full anæsthesia was induced and maintained, yet the obstruction was not in the least overcome or affected, and even after general or somatic death the steel-like band remained apparently, and certainly practically, as persistently contracted as before. And this has been the history of nearly all these cases as yet observed. In Dr. Baltzell's case, one of the first on record in this country, occurring in Frederick County, Md., the woman had been twice freely bled, forceps had been repeatedly used, version had been attempted, and yet at the autopsy, 4 hours after death, the constricting ring was still hard, firm and unyielding.

This whole case is replete with interest and at every point. In the first place, up to 1882, the date of Dr. Smith's paper, he had found on record and authentic 33 cases, occurring in 30 women; in the second place, they have all or nearly all occurred in the hands of obstetricians of acknowledged skill and experience, very many of them having been under the observation of recognized masters in the art; in the third place, they have resisted very generally the most enlightened and persistent efforts at relief; in the fourth place the mortality has proved appalling. In 33 labors of 30 women 8 women and 25 children were lost, 3 women dying undelivered; of the whole number 7 were primiparæ, of whom 4 died.

The mere mention of some of those who have treated and recorded these cases suffices to show that everything which intelligent knowledge could effect was done. Drs. Hosmer and Stone of New England, Dr. T. A. Reamy of Cincinnati, Prof. Elliott of New York, Prof. J. Y. Simpson of Edinburgh, Drs. Tabor Johnson and T. C. Smith of Washington, D. C., Angus McDonald, and others. Forceps, version, opium to full extent, chloroform to surgical anæsthesia repeated and continued, craniotomy, have all been resorted to, and all with like result. And even where these means or greater part had been used, in more than one case, as I have said, the unyielding bone-like or steel-like structure has persisted even after death. The character and history of these cases has led to their being termed by Harris "Tetanoid Falciform Contraction of the Uterus," by Smith and Hosmer," "Ante-Partum Hour-glass Contraction of the Uterus." Their whole history is that of spastic contraction. This could only occur in muscular structure, and the question immediately arises as to its seat, whether, as believed by Hosmer and others, it is at the internal os, or whether, as more generally believed, it is in the muscular structure above that point. In the majority of cases the observers have stated it was at a point too high to correspond to the internal os, even at times by those who have doubted whether any such condition as post-partum hour-glass contraction ever exists. So acute and experienced an observer as Dr. Reamy says of one of his cases: "It seemed certain that the band of constriction did not pursue a direction corresponding to the os internum." In the case of Dr. Arnold, of Roxbury, Mass., "the constriction was about the upper third of the uterus, encircling the child and inclosing its hips and lower extremities." Dr. Gray states of his case, seen in consultation, "the uterine contraction was not that of the os uteri but existed above it. In this he could not be mistaken."

One of the most important and satisfactory instances is that of Dr. P. R. Shaver, of Stratford, Ontario. It was a case of twins, and he states and reiterates "that one child and its placenta were distinctly contained in the lower compartment, the second child and its placenta in the second and upper compartment above the stricture." Now, as a child and placenta could not have been contained and going on to term in the cervical cavity, the seat of constriction could not have been at the internal os. In the case I now report the constriction was certainly too high for the internal os. Where then could it be?

These cases differ from those of Bandl, for the history and etiology of which we owe him so much in connection with rupture of the uterus. In cases of rupture almost uniformly we find some mechanical resistance to the progress of the child, in advance of it, by contracted pelvis, by mal-presentation of fœtus, by too large size physiological or pathological of the latter; we find the lower uterine segment elongated excessively, thinned until almost like paper, stretched and distended to the utmost and ultimately ruptured. In these the obstruction is above the lower uterine segment, clasping some part of the child, the inferior segment itself soft, yielding, not thinned, not stretched, and rupture not occurring. I cannot see that it can occur elsewhere than in the musculature of the body of the uterus, entirely above the os uteri internum, and constituting a true ante-partum hour-glass contraction. If then it is in the body of the uterus, can we determine its special seat and mode of production? Dr. Smith quotes Dr. Parish, of Philadelphia, in a discussion of post-partum hour-glass contraction in 1879 as saying: "Professor Hélic has demonstrated the existence of bundles of fibres running obliquely from either side of the body anteriorly and posteriorly to the fundus."* Professor Hélic states that a spasmodic contraction of these fibres in one half of the uterus would divide the uterus into two compartments, one of these being that portion of the cavity into which either Fallopian tube opens, *i. e.*, "the infundibulum," and the other compartment being the rest of the uterine cavity. Professor Hélic states that he has twice verified this by most careful digital examination, and in each case had to dilate the closely constricted portion to remove the incarcerated placenta."

Continuing, Dr. Parish goes on and states: I have been informed by Dr. R. G. Curtin that, in Cæsarean section performed by himself, a sulcus, transverse in direction, was seen to come and go in the body of the uterus. This sulcus, in the external surface, was evidently due to contraction of bundles of transverse fibres in the middle of the body, for after the uterine incision had been closed there was noticed an irregular gaping of its edges at the time of the formation of the transverse groove. A spasmodic contraction of the fibres that produced this shallow furrow, could have undoubtedly produced a marked hour glass contraction of the body of the uterus. There was no abnormal alteration of tissues in the uterus. "This furrow seen by Dr. Curtin was evidently the so-called Ring of Bandl, which I have more than once felt and seen."

In the case of Sænger's operation by Dr. Neale in 1887, this furrow could be plainly seen through the abdominal walls by the most superficial observer. The oblique fibres so well described by Professor Hélic, will not account for this furrow, or for the condition to which I have called your attention. To probe this matter a little further, I must beg your patience for a time, while we examine a fragment of anatomical and physiological history which may throw some light upon it, and I am the more disposed so to do, as our text-books are not very clear upon these points, and as I know that the views, not only of students, but of practitioners, are too frequently obscure and their knowledge but partial, taking words for things.

Although as far back as the 17th century, some, as DeGraaff 1671, Verhagen 1701, Weitbrecht 1750, held that the cervix remained unchanged until the end of pregnancy, Roederer in 1753 asserted the expansion of the cervical canal, and shortening, advancing regularly from above downwards and commencing as early as the sixth month. This continued the prevailing doctrine until 1826, when Stoltz insisted that the cervix was unchanged in length until the last fifteen days of pregnancy, and then the internal os opens, the cervical canal dilates from above downwards, and the cervix is gradually effaced, softening beginning below and extending upwards, shortening beginning above and extending downwards. In 1862, I. E. Taylor, N. Y., made some important observations showing that the cervix did not shorten, at least in many instances, until the inception of labor. W. Braune, in 1872, followed by Bandl, in 1876, and A. Martin, 1877, returned to and revived the old doctrine of Roederer. Braune saw in the frozen cadaver of a woman dying during labor, the lower segment of the uterus very much thinned, and above this thinned portion, a determined wedge-like muscular ring projecting into the interior of the uterus, the ring of Bandl, which he locates at 4 to 6 inches above what is generally recognized as the ring of Müller. Now this ring of Müller is the os internum, while above this is the aforesaid wedge-like projection of Braune, or ring of Bandl, or the second os internum of Scanzoni, between the body proper and the lower segment of the uterus. Braune and Bandl teach that during the last ten weeks of pregnancy the lower segment of the uterus and the upper part of the cervix soften, the latter dilates and permits the entrance and descent into it of the lower part of the ovum, thus forming what has been termed the cervico-uterine canal of Braune, the wedge-like ring above being the real os internum, this dilated portion being the cervical canal at its upper part, and therefore that the cervix does shorten during pregnancy. Scanzoni, Müller, Barnes, Holst, Schroeder, Duncan, Taylor, Spiegelberg, Hoffmeier and others have proved that the os internum does not, as a rule, open before labor, nor the neck shorten. The latest examinations upon this subject are by Hoffmeier and Benckeiser, 1888, who fully sustain this view. They show by their sections of uteri unimpregnated and pregnant, that the uterus is divided into three parts of segments, the upper part of the body, the Barnes' zone, just above the neck and the cervix. They, I think, have demonstrated that the cervix, as a rule, does not open above or shorten until labor sets in, that above the os internum the lower zone is narrow in the unimpregnated organ, differing in histology and structure from the cervix, and from the upper part of the corpus. The cervix retains all its characteristics up to labor. In the latter months the lower zone thins, dilates, and between this and the upper zone is found the ring of Bandl. All these latter find the os internum evident at time of labor, and Bandl's ring entirely above it.

What then is this so-called ring of Bandl? It can only form at the junction of the thickened wall of the corpus uteri, with its middle or medullary layer, as described by Savage, with the lower segment which is so much thinner. Both the fundus and the lower uterine segment are materially thinner than the intermediate portion. The latter is often two or three times as thick as the lower segment. The effect of contraction then would be felt particularly in the distention of this lower segment. The tissues there are thinner, fibres are more nearly longitudinal, and therefore offer a weaker resistance. The weight of the ovum, when the woman is erect, presses upon this part, and the action of the abdominal muscles is transmitted in this direction. Barnes has worked out practically the same thing with regard to his cervical zone or zone of danger, in connection with the natural history of placenta prævia.

The ring of Bandl and of Braune is certainly not the internal os, and it can only be found and formed at and by the lower edge of the medulla, where this plexiform layer thins out and loses itself in the cervical segment. Barnes' specimens distinctly show this well defined lower segment, which is clearly distinguished from the body above by three features: 1. It is thinner. 2. The muscular structure is less marked. 3. It is less rich in vessels. This lower segment is divided from the body by a more or less marked ridge which goes all around the uterus, Bandl's ring. From this ring upwards the walls of the organ begin to be richer in vessels, the wide lacunæ of the middle layer begin, and corresponding to this line on the outer wall, large vascular trunks run in and out of the uterus. This boundary between the body of the uterus and its lower segment is easily felt in the living organ at the level of the pelvic brim, and has been called by Lahs "the pelvic brim ring or stricture." It can be felt on introducing the hand to practice version and often when the hand is introduced to remove the placenta. One again sees the difference in structure in the two portions, in well-developed uteri which have been some time steeped in alcohol, the middle medullary layer of the body of the uterus ending in a point downwards, between the inner and outer layers of the lower segment. The marked development of this lower segment begins at a date not exactly determined, but the walls are observed to become softer towards the seventh month. The title, "Pelvic brim ring of Lahs" would not be very precise, the name of Bandl's or Braune's ring would not express the truth as they both consider it the os internum. The better name would be the contraction ring of Schroeder, or the retraction ring of Lusk, as it is at the junction of the corpus with the lower or cervical zone, at the level or line where the middle, thick or medullary layer ceases, and when in obstructed labor it becomes largely displayed as when rupture is threatened, when it is evident to palpation through the abdominal walls, and even to the sight, as I have seen it, and more than once, it is clearly due to this extent to the contraction and retraction of the upper thicker walls of the body of the uterus. In the light, then, of thorough examination by the most competent observers, and of practical experience, we are forced to the conclusion that this seat of the constriction is the lower edge of the medulla, the so-called ring of Bandl. As yet we are not able to trace or determine the disposing cause for its appearance. It is true that in a considerable proportion of the cases recorded the waters have been early, and in some long evacuated. But we know how often this occurs without such result, and in many of these cases it did not present. In Bandl's cases of rupture or threatened rupture, to which he particularly and so intelligently called attention, this constriction or hour-glass contraction was situated at the same point, the wedge or ring was produced at the same site and in the same manner, and there was always an obstruction before the presenting part of the child, when of course the contraction of the medullary and fundal portions of the uterus, with their retraction, must produce this effect; and the cause is evident and at once appreciated. In this category, however, there is generally no such obstruction and the producing cause, so far, eludes our grasp. It is certainly, however, important to learn and determine its true seat and character. Nor is this question one of simple, theoretical interest, but one of the utmost practical import as to the treatment.

With the inefficiency of the treatment heretofore employed the question would very naturally arise, and it has been suggested by Harris and Hosmer, whether we should not resort to Cæsarean section (Sänger's). If we take it to be at the internal os, this almost settles the question at once, as it would violate one of the fundamental laws of that operation; to respect, in our incisions, the cervix.

Apart from this, however, we have the difficulty and delay in diagnosis. The only general symptom in these cases, has been the early discharge of the liquor amnii, by no means followed necessarily or usually by this result. After this we have only the delay, until by this we are led to attempt extraction by one or another mode. Here, however, would come into strong relief the precious results of systematic external exploration by palpation, so useful, so pregnant with information, unfortunately so systematically neglected. If by palpation we detect Bandl's ring we know there is danger imminent, we know we cannot longer delay and wait, we are told at once that the period of expectancy has passed. It may portend rupture, it may be this hour-glass contraction, and in either case we must take the labor and at once into our own hands. But upon this external examination alone we should not found our absolute and special decision. This could only be done upon thorough internal exploration and the detection of the constricted ring. But trusting to internal exploration alone, we find the head movable at the superior strait, (there were only two cases of breech presentation), the os soft and dilatable, the cervix not thinned nor stretched, and almost intuitively and instinctively the practitioner would apply the forceps and then only learn the impossibility of extraction. The hand then introduced beyond the head, probably, if the obstetrist were ignorant of this condition, with the intent to turn, would find the fatal steel-like band. If the attendant be aware of the possibility of this accident, if by palpation he discovers the Contraction ring or Retraction ring, he would at once pass the hand above this presenting part and if he finds the Ring learn at once with what he has to deal. Now if this were at the os internum Cæsarean section would be almost forbidden, or at least its hazards would be vastly increased; if it be found situated where I have stated, and where I am sure it is with the inefficiency of all the means heretofore employed, I deem the modern Cæsarean section would find its place, as in cases of threatened rupture, where the obstruction in advance of the child could not otherwise be removed.

Up to 1882 Dr. Harris, whose opinion is of so much weight and value that I should hesitate long before differing with him, was disposed to take this view, but not until turning had been attempted.

But from all that he has later so well taught as to the propriety and superiority of early operation, and the great disadvantage of prolonged or active manipulation before the use of the knife, I think he would speak more positively as to the advisability of the early operation after a diagnosis were once certainly made.

This imperfect résumé of facts, for facts they are, upon this so important structure, justify us I think in coming to certain conclusions.

1. That a true "Hour-glass Contraction of the Body of the Uterus," (Smith and Hosmer) a "Tetanoid Falciform Contraction of the Uterus," (Harris) does occur during labor, and constitutes one of the most serious difficulties of the process.

2. That by palpation it may be detected in a large portion of these cases.

3. "Clinical facts demonstrate that the segment of the uterus below the stricture is in a relaxed condition, and only in exceptional cases is thinning thereof to be recognized," (Smith).

4. That from anatomical and post-mortem examinations most carefully conducted by most astute and reliable anatomists and pathologists, supported by clinical observations of the most searching character by obstetrists, acknowledged as masters of the art, this ring or contraction, the ring of Bandl, the contraction ring of Schroeder, or the retraction ring of Lusk, is not at the internal os uteri, but above the lower segment, the cervical zone of Barnes, at the lower edge of the body of the uterus, or of the medulla of Savage, and is produced by the contraction and retraction of this upper part of the body.

5. That this fact once established while we can not yet positively formulate their treatment, their mortality has been so large and the success of the modern Cæsarean section has been so great, that it speaks strongly in favor of the latter.

One word in conclusion in connection with the case of Dr. Wilson. I have been asked, whether in view of the history of this case, there might not have been a rupture of the uterus. Having this possibility in view, after my recognition of its character, I most carefully, in my manipulations, examined every part of the organ and can state positively that no such lesion existed.

Hæmorrhage certainly was not the cause of death. It may have been due to embolism, but in the absence of a post-mortem, we would not be justified in such an assertion.

In the 30 women whose cases were collected by Dr. Smith, there were three cases of contracted pelvis, one of shoulder presentation and one of fibroid before the child, partly filling the pelvis.

In only one case was the cervix thinned, and that was reported by Bandl, being a face presentation.

There was one case of rupture [case xv] but the reporter states, an amount of force was used which under other circumstances would be unwarrantable, and that external pressure was made over the head, the rupture being a bruised laceration at this point where presure had been made upon the head in the attempt at version.

ADDENDA.

It is of interest and importance to note that these various anatomical details have been worked out without reference to this special accident.

Since writing the above two interesting cases have come to my knowledge; the one at full term reported by Dr. S. T. Earle, of Baltimore, the other at six months by Prof. T. A. Ashby. One mother lost, and both children.

Results of new Cæsarean Section; Sänger; by Dr. Harris, Sept., 1886. *Medical News.*

In Germany 84 per cent. of women saved.

In Austria 50 per cent. of women saved.

In Italy 50 per cent. of women saved.

In France 100 per cent. of women saved, only two cases.

Potocki, in June, 1886, gives the following table of operations done at Dresden and Leipzig:

Ten by Leopold and his assistant Korn, and 6 by Sänger and his assistants Obermann and Donat. Fifteen recoveries for mothers—93.7 per cent.; 16 children living —100 per cent.

If we add to these 3 cases at Innsbruck, all of which were successful, we have 19 operations and only (1) one woman lost.

Caruso gives us the very latest data of the new Cæsarean operation: Up to October 1st, 1888, comprising 135 cases; six successful cases in addition, are known to Caruso, but the details necessary for publication were lacking.

The results are 74.44 per cent. of recoveries among mothers in all cases, and 91.73 per cent. recoveries among children.

The mother has three chances out of four, and her child nine out of ten for life with this operation. What a contrast with the results of these cases in this paper; in 36 cases occurring in 33 women, 10 mothers and 28 children were lost.

Dr. H. M. Wilson thanked Dr. Miltenberger for his exhaustive résumé. Some points connected with the case made it very peculiar. The surroundings were all satisfactory, and nothing occurred to suggest any danger until the failure to deliver by forceps. Then noticing the strange appearance of the face, the inhalation of chloroform was instantly suspended. She soon came from under its influence. Hypodermics of brandy were repeatedly administered, but without the slightest impression on the fatal collapse which ended life in from 30 to 40 minutes. During this brief time every effort was directed towards sustaining life, and no opportunity afforded for further efforts at delivery. In his study of this case no explanation of the sudden collapse is satisfactory, except embolism, and this is only a conjecture as no post-mortem was had. No ergot had been used and but little chloroform. Dr. Smith of Washington, who wrote concerning the case, thought rupture of the uterus might have taken place, but positive proofs to the contrary were adduced by Dr. Miltenberger. Tetanoid contraction of the uterus is happily infrequent, but the collapse attending the case under discussion renders it unique as far as his experience or reading extends.

Dr. John Morris said, that were it not for Dr. Miltenberger's most positive statement, he should consider this a case of ruptured uterus; it is in every particular a history of such condition. With the ring of Bandl he had been acquainted long before it had been so named; every obstetrician has met with it, but he has been accustomed to hear it called "the hour-glass contraction." He could not understand the death of this woman, nor the tetanoid contraction; such a condition he had never seen. She had no hæmorrhage, and was not exhausted. The only theory that he could advance was an embolism.

Dr. T. A. Ashby said: I have listened with great interest and profit to the paper read by Dr. Miltenberger. He has treated the subject so thoroughly that nothing has been left for discussion. The paper suggests an experience which corresponds with that related in the paper. Its recital in this connection will not be out of place. On the 10th of September, 1888, I was requested to go to the country to see the wife of a medical acquaintance living in Anne Arundel county. I was advised to come prepared to remove a retained fœtus. Upon reaching the house I found the patient, a primipara, about 22 years of age, in the following condition: Twenty-four hours previously, without known cause, labor pains came and she partly expelled a six months' fœtus. Her husband had attempted to remove the fœtus, but finding that he could not do so desisted, and called on me for help. I found the fœtus enclosed in the amnion, presenting with its head external to the vulva. Attempting to remove it, it was found firmly grasped by the uterus as if in a vise. The finger could be passed around the head and shoulders and up into the uterus, the cervix being open and dilatable. There was no opposition to its expulsion from the pelvic canal, but it was firmly caught around the abdomen by a tetanic contraction of the uterus at the ring of Bandl. In this condition it had remained for some hours, the patient having lost all expulsive power. In an attempt to remove the fœtus its head nearly parted from its body, and the uterus was drawn down to the vulvar opening. After some effort, I succeeded in removing the entire body, but the constriction ring contracted so firmly that the placenta was held in the uterine cavity. The finger would not overcome the constriction and it became necessary to introduce the curette and remove the placenta piecemeal. The patient bled profusely and came near perishing from hæmorrhage before I could remove the placental tissue and secure retraction of the organ. By the use of hot water, vinegar and, lastly, a dilute solution of Mônsel's solution, all bleeding was stopped and a successful recovery followed. The history of the patient presented no explanation as to the cause of premature separation, and for the anomalous condition of tetanoid contraction at the sixth month of gestation. The condition observed was strikingly similar to that reported by Dr. Miltenberger, in the case of Dr. H. M. Wilson, the exceptions being in the premature development of labor and the recovery of the mother. The fœtus presented evidences of having been dead several days. Why this tetanic condition of the uterine muscles should have continued for so many hours (about 24) I am unable to explain. The amnion had been exposed external to the vulva opening long enough to assume a parchment condition and undergo partial decomposition.

Dr. G. W. Miltenberger, in reply to Dr. Morris, recalled Baltzell's case, in which the rigidity still continued at the autopsy, four hours after death. Such occurrences are common. Organic life still persists after the entire cessation of the life of relation. The hair and nails grow after general or somatic death. Posthumous births show the same thing. Leroux felt the uterus contract fifteen minutes after the death of the mother. Osiander saw it contract as during life in making a post-mortem Cæsarean section. As to the detection of the so-called Bandl's ring by palpation, he has more than once not only felt but seen it. It is produced by contraction and retraction of the upper part of the corpus-uteri. In reply to Dr. H. M. Wilson's question, as to the probability of turning in the given case during life, he said that over and over again the best men had been foiled in attempting version in these cases, and in this case of Dr. Wilson's he was convinced that without inquiry he could not have succeeded during life. He then gave a full description of the ring of Bandl, and of the inferior segment of the uterus, the conical zone, or zone of danger of Barnes, and said (a matter of some interest in connection with his paper), that the softened zone which constituted Hégar's sign of early pregnancy was of course this same thin inferior segment, between the firm cervix below and the lower edge of the medulla of Savage above.

ALBUMINURIA IN THE PREGNANT FEMALE.

Dr. G. Lane Taneyhill exhibited a test tube so heavily loaded with albuminous urine, 90 per cent., that lying in a horizontal position the substance was not displaced, which, he remarked, was voided three days since by a woman aged 36, light hair, of low stature, stout, who had severe headache with dropsical effusion, so extensive as to involve the face, and who expects to be confined in a few days. She has had two children, the youngest being 15 years old. He is administering 5 grains of benzoic acid with 10 grains of bicarbonate of potash every three hours, and produces free catharsis by large doses of epsom salts, every forty-eight hours. He had used this combination in the obstetric wards of Bellevue Hospital, in 1868, in similar cases with considerable degree of success, and had continued the same practice for the last twenty years in all cases when called in ample time to institute medical treatment. He understood that it was held by Golding Bird that benzoic acid exhibited in cases of albuminuria possessed the power of obviating the toxæmic state by preventing the waste of albumin and removing the urea. While he agreed with Dr. Opie that special medication was not apt to be of much avail where the albuminuria depended solely upon the mechanical pressure of the gravid uterus, yet he was inclined to believe that by this eliminating process vigorously prosecuted, he had averted cases of eclampsia; that a sample of this same woman's urine, tested to-day, showed some decrease in the amount of albumin and the headache had disappeared. Comprehending the gravity of the situation he interrogated the society regarding the treatment most applicable in a case of albuminuria previous to confinement.

Dr. H. M. Wilson remarked, that the amount of albumin indicated a very serious condition. He advised a continuance of the saline treatment, preferring potas bitartras. When labor commenced, the first indication of brain sympathy, such as twitching, should be the signal for chloroform and the forceps. Indeed, under the circumstances surrounding this case, he should think prudence would suggest the termination of labor at the very earliest moment and by the readiest means.

